# A Natural Compound Containing a Disaccharide Structure of Glucose and Rhamnose Identified as Potential N-Glycanase 1 (NGLY1) Inhibitors

**DOI:** 10.3390/molecules28237758

**Published:** 2023-11-24

**Authors:** Ruijie Liu, Jingjing Gu, Yilin Ye, Yuxin Zhang, Shaoxing Zhang, Qiange Lin, Shuying Yuan, Yanwen Chen, Xinrong Lu, Yongliang Tong, Shaoxian Lv, Li Chen, Guiqin Sun

**Affiliations:** 1 School of Medical Technology and Information Engineering, Zhejiang Chinese Medical University, Hangzhou 310053, China; liuruijie523@163.com (R.L.); yylin995@163.com (Y.Y.); zhangyuxin0005@163.com (Y.Z.); shaoxingz@163.com (S.Z.); linqiange0709@163.com (Q.L.); 2 School of Basic Medical Sciences, Zhejiang Chinese Medical University, Hangzhou 310053, China; gjj0824@126.com; 3 Department of Clinical Laboratory, Jiaxing Maternity and Child Health Care Hospital, Jiaxing 314001, China; yuansy1126@163.com; 4 Central Laboratory, Ningbo Hospital, Renji Hospital, Shanghai Jiao Tong University School of Medicine, Ningbo 315336, China; wlgwzyy@163.com; 5 Key Laboratory of Medical Molecular Virology (MOE/NHC/CAMS), School of Basic Medical Sciences, Fudan University, Shanghai 200032, China; 20111010058@fudan.edu.cn (X.L.); 22111010078@m.fudan.edu.cn (Y.T.); 23111010078@m.fudan.edu.cn (S.L.)

**Keywords:** N-glycanase 1 (NGLY1), NGLY1 inhibitor, natural compound, structure-based virtual screening

## Abstract

N-glycanase 1 (NGLY1) is an essential enzyme involved in the deglycosylation of misfolded glycoproteins through the endoplasmic reticulum (ER)-associated degradation (ERAD) pathway, which could hydrolyze N-glycan from N-glycoprotein or N-glycopeptide in the cytosol. Recent studies indicated that NGLY1 inhibition is a potential novel drug target for antiviral therapy. In this study, structure-based virtual analysis was applied to screen candidate NGLY1 inhibitors from 2960 natural compounds. Three natural compounds, Poliumoside, Soyasaponin Bb, and Saikosaponin B2 showed significantly inhibitory activity of NGLY1, isolated from traditional heat-clearing and detoxifying Chinese herbs. Furthermore, the core structural motif of the three NGLY1 inhibitors was a disaccharide structure with glucose and rhamnose, which might exert its action by binding to important active sites of NGLY1, such as Lys238 and Trp244. In traditional Chinese medicine, many compounds containing this disaccharide structure probably targeted NGLY1. This study unveiled the leading compound of NGLY1 inhibitors with its core structure, which could guide future drug development.

## 1. Introduction

**Figure 1:**
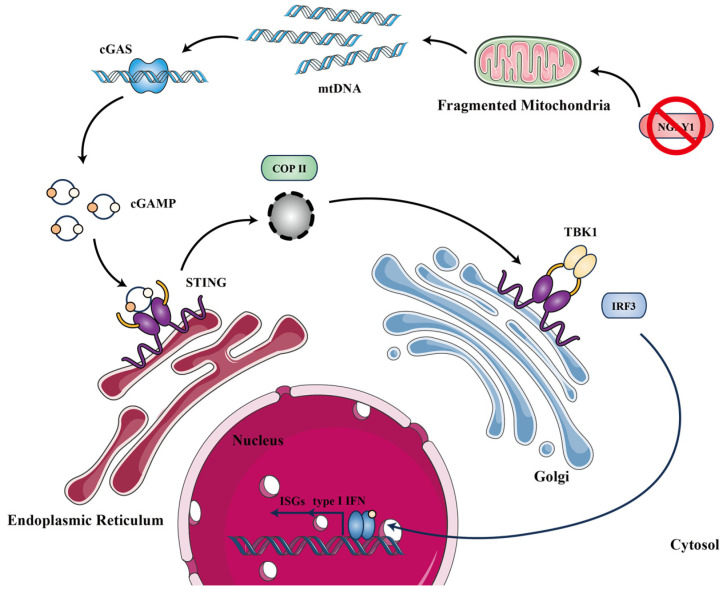
Schematic diagram of antiviral effect by downregulating NGLY1 expression. NGLY1 is an essential enzyme involved in deglycosylation of misfolded glycoproteins. Knocking out the expression of NGLY1 leads to severe mitochondrial fragmentation, causing the release of mitochondrial DNA into the cytoplasm. Innate immune detection of self-DNA by the DNA sensor cGAS activates downstream STING-TBK1-IRF3 signaling cascade, inducing the expression of type I interferons (IFN) and IFN-stimulated genes (ISGs), effectively promoting an antiviral response [10]. mtDNA: mitochondrial DNA; cGAS: cyclic GMP–AMP synthase; cGAMP: 2′3′ cyclic GMP–AMP; STING: stimulator of interferon genes; COP-II: coatomer protein complex II; TBK1: TANK-binding kinase 1; IRF3: interferon regulatory factor 3; type I IFN: type I interferons; ISGs: IFN-stimulated genes.

N-Glycanase 1 (NGLY1) is a de-N-glycosylating enzyme that catalyzes the hydrolysis of the amide bond between the proximal N-acetylglucosamine (GlcNAc) residue and the Asn side chain to which it is attached, removing N-glycans from glycosylated proteins in the cytosol [1,2,3]. Discovered in 1993, NGLY1 is known to participate in clearing misfolded glycoproteins during the process of glycoprotein synthesis through the endoplasmic reticulum (ER)-associated degradation (ERAD) pathway [4]. NGLY1 deficiency leads to disrupted ERAD function. Histological analysis of *Ngly1^−/−^* rats demonstrated cytoplasmic ubiquitinated protein accumulation in neurons of the thalamus and spinal cord [5]. In 2014, it was reported that NGLY1 deficiency could lead to NGLY1-congenital disorder of deglycosylation (CDDG), a disease characterized by developmental delay, intellectual disability, absence of or reduced tears and sweating, abnormal liver function, and motor dysfunction [6,7,8]. It was noteworthy that NGLY1-CDDG patients were less susceptible to viral infections [9]. Additionally, it has been reported that generated *Ngly1* knockout (*Ngly1^−/−^*) murine embryonic fibroblasts (MEFs) inhibited vesicular stomatitis virus (VSV) replication [10]. siRNA knockdown of NGLY1 or Z-VAD-FMK (benzyloxycarbonyl-Val-Ala-Asp-fluoromethyl ketone) inhibits NGLY1, restricting the infection of enterovirus 71 (EV71) and coxsackievirus A16 (CA16) in RD cells [11]. Yang et al. [10] also found NGLY1-deficient human and mouse cells, resulting in severely fragmented mitochondria and the activation of cGAS–STING pathways, leading to elevated IFN-stimulated genes (ISGs). A schematic diagram is shown in Figure 1. NGLY1 represents a potential drug target for antiviral therapy. NGLY1 inhibitors screening is an important pathway for discovering potential antiviral lead compounds. Currently, Z-VAD-FMK is reported to inhibit NGLY1 irreversibly by covalently binding to the active site, but it also inhibits pan-caspase [12,13]. Hence, the pursuit of milder NGLY1 inhibitors holds critical significance.

NGLY1 is a unique protein that plays a role in the ERDA pathway as well as in the activation of key signal proteins such as Nrf1 [3], and its regulation may provide new insights into health and disease. Although efforts have been made on compounds for the regulation of glycosylation from molecule compounds [13] and natural compounds [14], as well as those obtained through synthetic methods [15,16], reports of natural compounds targeting NGLY1 are rare. Commonly used natural compounds from plants, animals, and microorganisms have various beneficial effects on our health, and natural compounds have fewer side effects and are, thus, advantageous for therapeutic purposes [17,18,19]. One example is artemisinin isolated from artemisia annua L., which has been used for the treatment of malaria [20]. Similarly, paclitaxel, isolated from taxus brevifolia, has been used to treat some malignancies, such as lung, breast, and pancreas cancers [21]. The natural compound has been found to exhibit various effects. Yang et al. [22] found that the natural compound green tea polyphenols exhibited inhibitory effects on proteasomes, suggesting their potential applications in the prevention and treatment of cancers. It also has been reported that two substituted derivatives of the natural compound salacinol showed marginal activity against O-GlcNAcase [23]. Furthermore, many traditional Chinese medicines are known for their heat-clearing and detoxifying properties [24], and natural compounds isolated from these medicines exhibit potential antiviral effects. It has been reported that the natural compound flavonoids acted at different stages of viral infection, such as viral entrance and replication [25]. They had the potential to impede the attachment and entry of viruses into cells, disrupt various stages of viral DNA replication, protein translation, and poly-protein processing [26].

Molecular docking, based on a protein structure, is a method employed to predict the binding modes and affinity of ligands within complexes [27,28,29]. This approach is crucial in screening ligands within chemical libraries, providing valuable insights into their interactions with biological targets. Consequently, molecular docking studies have emerged as highly accurate and powerful tools for analyzing the interactions between active compounds and potential targets [30,31].

In this study, we employed computer-aided drug screening methods to identify three compounds capable of inhibiting NGLY1 from a library of 2960 natural compounds. The inhibitory effects were further validated through an electrophoretic mobility shift assay. They were isolated from monomers of traditional Chinese medicine for clearing heat and detoxification. We also found that the core structure of NGLY1 inhibitors consists of a disaccharide structure composed of glucose and rhamnose. These compounds might act as lead compounds of NGLY1 inhibitors and may have antiviral effects.

## 2. Results

### 2.1. Establishment of a Screening Method for Targeting NGLY1 Lead Compounds

**Figure 2:**
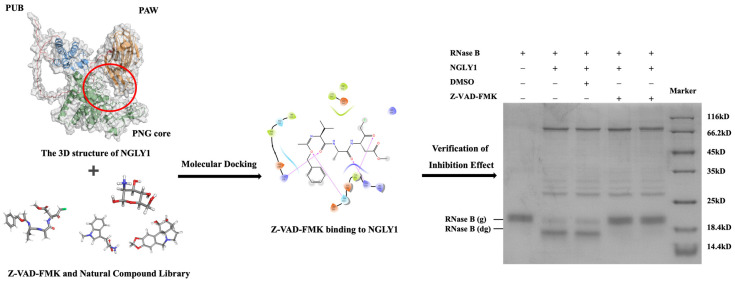
The screening method for targeting NGLY1 lead compounds. Red circle: Location of the receptor pocket. Red arrow: Location of the NGLY1. RNase B (g): N-glycosylated RNase B (NGLY1 is inhibited); RNase B (dg): N-glycosylated RNase B (NGLY1 is not inhibited).

Molecular docking plays a pivotal role in the drug screening process. In this study, we developed a natural lead compound screening method targeting NGLY1. By predicting the three-dimensional structures of NGLY1, we performed molecular docking with 2960 natural compounds derived from plants, animals, or microorganisms. To identify potential inhibitors, we compared the docking scores of these compounds with that of the known NGLY1 inhibitor Z-VAD-FMK. Compounds exhibiting docking scores lower than −4.56 were then selected as primary screening compounds. The enzyme NGLY1 exhibits the same cleavage effect as PNGase F (Figure S1), and the inhibitory effects of the primary screening compounds were validated through an electrophoretic mobility shift assay. We validated the feasibility of this screening method using Z-VAD-FMK, as shown in Figure 2.

The lower binding affinity energy indicates stronger binding affinity. Among the 2960 compounds screened, a total of 215 compounds exhibited docking scores lower than −4.56. Considering the known biological activities of these compounds, we further narrowed down the selection to 17 compounds associated with glycosylation or possessing anti-inflammatory, antioxidant, and anti-tumor properties (Table S1). Among these 17 compounds, 13 of them contained glycan structures. Therefore, we speculated that compounds with glycan structures might have a higher affinity with NGLY1.

### 2.2. Verification of Inhibitory Effect of Primary Screening Compounds

**Figure 3:**
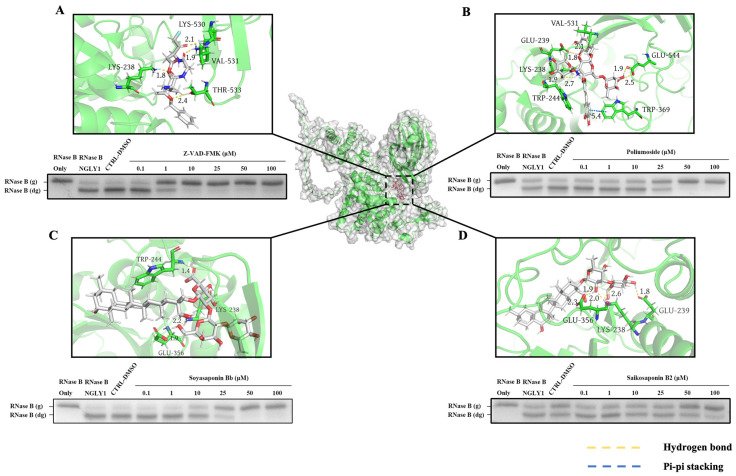
Analysis of interaction mode between NGLY1 inhibitors and NGLY1 protein, and NGLY1 inhibitors’ mediated inhibition of NGLY1 activity were tested at various concentrations using the electrophoretic mobility shift assay. (**A**) Sites of Z-VAD-FMK binding to NGLY1 and Z-VAD-FMK mediated inhibition of NGLY1 activity. (**B**) Sites of Poliumoside binding to NGLY1 and Poliumoside Bb mediated inhibition of NGLY1 activity. (**C**) Sites of Soyasaponin Bb binding to NGLY1 and Soyasaponin Bb mediated inhibition of NGLY1 activity. (**D**) Sites of Saikosaponin B2 binding to NGLY1 and Saikosaponin B2 mediated inhibition of NGLY1 activity. RNase B (g): N-glycosylated RNase B (NGLY1 is inhibited); RNase B (dg): N-glycosylated RNase B (NGLY1 is not inhibited).

The inhibitory effects of primary screening compounds were validated through the electrophoretic mobility shift assay. Specifically, Poliumoside, Soyasaponin Bb, and Saikosaponin B2 demonstrated effective inhibition of NGLY1 activity. The minimum inhibitory concentrations of Z-VAD-FMK, Poliumoside, Soyasaponin Bb, and Saikosaponin B2 were found to be 1 mM, 25 mM, 25 mM, and 50 mM, respectively (Figure 3). It is noteworthy that all three compounds were isolated from traditional heat-clearing and detoxifying Chinese herbs. The strongest dockings of NGLY1 inhibitors and their binding sites, interactions, and distances, are shown in Figure 3 and Table 1. NGLY1 inhibitors mainly bind to amino acids, such as Lys238, Glu239, Trp244, and Glu356, through hydrogen bonding.

### 2.3. The Analysis of Inhibitory Mechanism of NGLY1 Inhibitor

**Figure 4:**
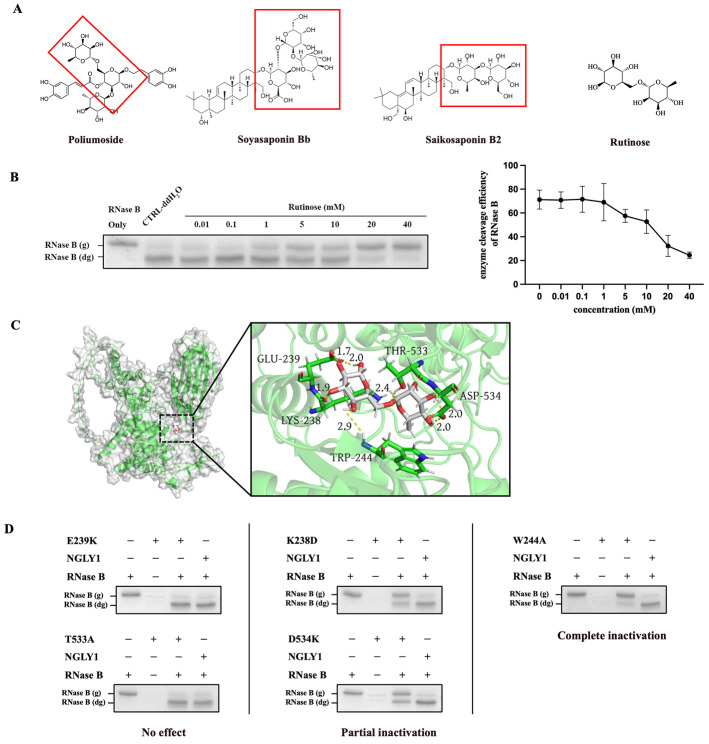
The inhibitory effect of Rutinose as a core structure of Poliumoside, Soyasaponin Bb, and Saikosaponin B2 on NGLY1. (**A**) Chemical structure of Poliumoside, Soyasaponin Bb, Saikosaponin, Rutinose. (**B**) Rutinose mediated inhibition of NGLY1 activity. Further quantification of the enzyme cleavage results was performed using Image J (Version 2.1.0) software. (**C**) Sites of Rutinose binding to NGLY1. (**D**) The activity of NGLY1 mutants. RNase B (g): N-glycosylated RNase B (NGLY1 is inhibited); RNase B (dg): N-glycosylated RNase B (NGLY1 is not inhibited). Red box: The glycan structure of the compound.

We compared the chemical structures of Poliumoside, Soyasaponin Bb, and Saikosaponin B2 (Figure 4A) and found that Poliumoside contains d-glucose and l-rhamnose, Soyasaponin Bb contains d-galactose (an enantiomer of d-glucose) and l-rhamnose, and Saikosaponin B2 contains d-glucose and d-fucose (an enantiomer of l-rhamnose). The results illustrated that the natural compounds with a core disaccharide structure of (d-glucose/d-galactose) and (l-rhamnose/d-fucose) may have inhibitory effects on NGLY1. We identified a compound, Rutinose (Figure 4A), which is a disaccharide structure containing d-glucose and l-rhamnose. The electrophoretic mobility shift assay showed that Rutinose inhibited NGLY1 in a dose-dependent manner at high concentrations (Figure 4B). As Poliumoside exhibited the highest potency, and the activity of Rutinose was confirmed, we utilized the disaccharide structure containing glucose and rhamnose as the core structural element for the NGLY1 inhibitor in this study.

The molecular docking results between Rutinose and NGLY1 showed five binding sites, Lys238, Glu239, Trp244, Thr533, and Asp534 (Figure 4C). Mutations were added to each of the five binding sites, and the results showed that E239K and T533A had no effect on NGLY1 activity, while K238D and D534K mutations led to the partial inactivation of NGLY1. The W244A mutation resulted in complete inactivation of NGLY1 (Figure 4D). Lys238, Trp244, and Asp534 were important catalytic sites of NGLY1. Poliumoside, Soyasaponin Bb, and Saikosaponin B2 might inhibit NGLY1 by binding to Lys238 or Trp244.

### 2.4. The Inhibitory Effects of Rutinose as the Core Structural Element for NGLY1 Inhibitor

**Figure 5:**
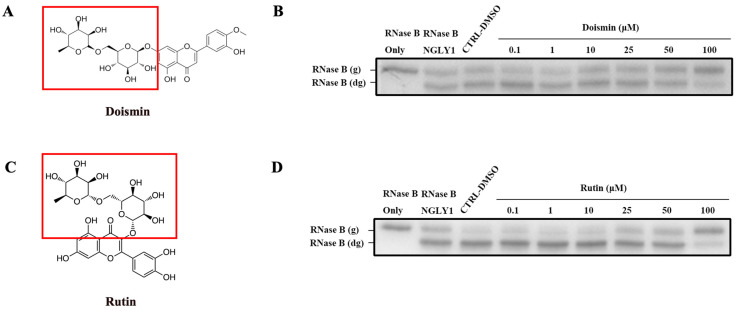
Doismin and Rutin mediated inhibition of NGLY1 activity were tested at various concentrations using the electrophoretic mobility shift assay. (**A**,**C**) Chemical structure diagram of Diosmin and Rutin. (**B**) Diosmin and (**D**) Rutin mediated inhibition of NGLY1 activity were tested at various concentrations using the electrophoretic mobility shift assay. RNase B (g): N-glycosylated RNase B (NGLY1 is inhibited); RNase B (dg): N-glycosylated RNase B (NGLY1 is not inhibited). Red box: The glycan structure of the compound.

To validate Rutinose as a core structure for NGLY1 inhibition, we identified two other compounds, Doismin and Rutin, which also contain D-glucose and L-rhamnose in their glycoside portion (Figure 5A,C). Through in vitro enzymatic assays, they were found to inhibit NGLY1 activity as well (Figure 5B,D), but their minimum inhibitory concentration was different. Therefore, we hypothesized that the glycoside composed of glucose and arabinose played a major inhibitory role but exhibited varying inhibitory effects due to different saponin ligands.

### 2.5. Toxicity Analysis

To ensure the safety of candidate drugs, toxicity testing of compounds was an important step. Calculation-based electronic toxicity measurement is widely utilized due to its accuracy and accessibility, providing information on any natural compound [32]. In order to determine the toxicity and adverse effects of the four NGLY1 inhibitors, we used the freely available testing tool ADMETlab 2.0 [33,34]. The website evaluates several toxicological parameters, such as human hepatotoxicity (H-HT), drug-induced liver injury (DILI), skin sensitization, carcinogenicity, eye corrosion, and eye irritation (Table 2). According to ADMETlab 2, Z-VAD-FMK and Poliumoside were highly probable to cause a drug-induced liver injury, with Poliumoside also posing a potential risk of skin allergy. Conversely, Soyasaponin Bb and Saikosaponin B2 exhibited a lower probability of toxicities.

**Figure 6:**
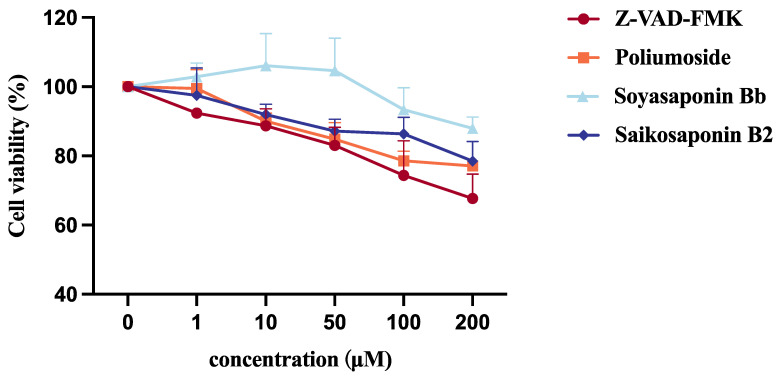
CCK8 analysis of HEK 293T cells treated with NGLY1 inhibitors for 24 h.

Subsequently, we also performed CCK-8 assays of HEK293T cells treated with four NGLY1 inhibitors. After 24 h of compound exposure, Soyasaponin Bb did not significantly impact cell viability and the impact of Poliumoside, Soyasaponin Bb, and Saikosaponin B2 on cell viability was less than that of Z-VAD-FMK (Figure 6). In summary, the compound Soyasaponin Bb demonstrated a higher safety profile compared to Z-VAD-FMK, whereas Poliumoside was predicted to have potential compound toxicity.

The results were predicted by the ADMETlab 2.0 website (https://admetmesh.scbdd.com (accessed on 10 October 2023)), but the actual toxicity of the drugs should be determined through real experiments.

## 3. Discussion

In this study, we screened three natural compounds that can inhibit NGLY1 activity. Leveraging the predictive structure of NGLY1, we conducted computational virtual screening using a natural compound library. In the initial round of screening, seventeen natural compounds were evaluated, leading to the identification of three promising NGLY1 inhibitors: Poliumoside, Soyasaponin Bb, and Saikosaponin B2. An intriguing revelation emerged upon comparing the chemical structures of these inhibitors. Remarkably, we observed a disaccharide structure composed of glucose and rhamnose, which had been proven to be effective in inhibiting NGLY1 and could serve as a core structural element for NGLY1 inhibitors. This core disaccharide structure could also serve as a lead structure for the design and development of NGLY1 inhibitors. Based on the molecular interaction analysis, we proposed that Lys238, Trp244, and Asp534 are important catalytic sites of NGLY1. Our findings suggest that NGLY1 inhibitors likely impede its function by binding to Lys238 or Trp244. Consequently, during the screening process, it was crucial to focus on compounds that interact with Lys238 and Trp244, as they may serve as a basis for subsequent drug screening for NGLY1 inhibitors.

Comparatively, Poliumoside, Soyasaponin Bb, and Saikosaponin B2 demonstrated less potent inhibitory effects on NGLY1 when compared to Z-VAD-FMK. However, it is worth noting that these compounds are natural products derived from plants with fewer drug-related side effects. We conducted preliminary toxicity predictions using ADMETlab 2.0 website (https://admetmesh.scbdd.com (accessed on 10 October 2023)) and assessed their effects on cellular viability for the three specific compounds, and found that the compounds Soyasaponin Bb demonstrated higher safety profiles compared to Z-VAD-FMK, whereas Poliumoside was predicted to have potential compound toxicity. Further research is necessary to investigate the impact of these compounds on intracellular NGLY1 activity within cells and to conduct metabolomics-based toxicological research, focusing on aspects such as nephrotoxicity, hepatotoxicity, cardiotoxicity, and central nervous system toxicity [35,36,37].

It has been reported that NGLY1 deficiency could upregulate the expression of ISGs, enhancing the body’s innate antiviral capabilities [10,38]. Consequently, NGLY1 inhibitors have the potential to complement traditional antiviral drugs by contributing to an enhanced antiviral response. NGLY1 inhibitors have demonstrated not only antiviral properties but also significant implications in cancer treatment. In the treatment of blood cancers like multiple myeloma (MM) and mantle cell lymphoma (MCL), proteasome inhibitors play a crucial role [39,40]. However, resistance to protease inhibitors primarily arises from the upregulation of proteasome subunit (PSM) levels, with the expression of the PSM gene being regulated by the transcription factor Nuclear Factor, Erythroid 2 Like 1 (NFE2L1 or Nrf1) [39,41]. Importantly, Nrf1 is an N-glycosylated transmembrane protein, and its activation process involves the participation of NGLY1 [42,43]. Tomlin et al. [3] found that inhibiting NGLY1 in cultured cells disrupts Nrf1 function, enhancing the cytotoxicity of protease inhibitors. Notably, NGLY1 is highly expressed in certain human cancer cells, such as melanoma and ovarian cancer, while being low in their corresponding normal tissues like skin and ovary [44]. It has also been reported that the downregulation of NGLY1 results in melanoma cell death and a slowdown in tumor growth [45]. Compounds, like Poliumoside, Soyasaponin Bb, and Saikosaponin, exhibit promising therapeutic potential in various tumor diseases, including melanoma, multiple myeloma, and acute lymphocytic leukemia.

Our future work will encompass a comprehensive assessment of the broad-spectrum antiviral properties associated with NGLY1 inhibitors, employing various types of viruses for thorough evaluation. Additionally, we will also investigate the potential therapeutic utility of NGLY1 inhibitors in the treatment of melanoma, multiple myeloma, and acute lymphocytic leukemia. Current efforts are concentrated on scaffold optimization to refine the molecular structure of the inhibitors, aiming to enhance their inhibitory potential. Dubbu et al. [15] utilized the Prins cyclization to synthesize 2-Deoxy-β-C-aryl/alkyl glycosides, while Chennaiah et al. [16] synthesized vicinal 1,2-azidoacetates catalyzed by TMSOTf, offering a method for structural optimization of NGLY1 inhibitors.

## 4. Materials and Methods

### 4.1. Molecular Docking

A three-dimensional structure of NGLY1 was predicted online using AlphaFold2 based on the NGLY1 amino acid sequence (https://colab.research.google.com/github/sokrypton/ColabFold/blob/main/AlphaFold2.ipynb?pli=1#scrollTo=kOblAo-xetgx (accessed on 10 January 2022)). The SDF format structures of the natural compound library consist of 2960 compound structures’ information obtained from TargetMol (Boston, MA, USA).

NGLY1 consists of three domains: PUB, PNG Core, and PAW. The PNG Core plays the primary catalytic role. Preliminary experiments have shown that mutations of amino acids Trp236, Trp244, Cys283, Leu318, Cys355, Glu356, and Asp386 in the PNG Core domain result in protein inactivation. The receptor pocket was generated with the aforementioned amino acids as the center (x = 5.47, y = 4.28, z = 6.22). The receptor pocket was set as a cubic grid with a side length of 20 Å. Semi-flexible docking was employed. Structural analysis of the docking results was performed using PyMOL (2.5.2) software.

### 4.2. Construction of NGLY1 Mutant

Plasmid pET28a-NGLY1 (WT) was preserved by our laboratory. All NGLY1 mutants were generated using the reverse complement primers for PCR (Takara, Beijing, China) with the template of pET28a-NGLY1 (WT) plasmid. The constructed pET28a-NGLY1 (WT) plasmid and pET28a-NGLY1 (mutant) plasmid were transformed into E. coli BL21 (DE3) (Tiangen, Beijing, China) separately for protein expression. The primers can be found in Table 3.

### 4.3. Protein Expression and Purification of NGLY1 and NGLY1 Mutant

Escherichia coli BL21(DE3) cells harboring the NGLY1 and NGLY1 mutant expression plasmid were grown at 37 °C in a Luria-Bertani (LB) medium containing 50 μg/mL kanamycin. When the culture reached OD600 of 0.6–0.8, 1 mM isopropyl β-d-thiogalactoside (IPTG) (Sangon Biotech, Shanghai, China) was added to the medium and incubated further for 12 h at 28 °C.

The frozen cells were resuspended in a lysis buffer (20 mM Tris, 300 mM NaCl, and 10 mM imidazole; pH 7.4) and then disrupted by sonication, and insoluble materials were removed by centrifugation at 12,000 rpm for 30 min at 4 °C. The supernatant was loaded onto a Ni-NTA resin column (1 mL bed volume, Thermo Scientific, Walthamm, MA, USA) and incubated for 30 min at 4 °C. The column was washed with lysis buffers and wash buffers (20 mM Tris, 300 mM NaCl, and 25 mM imidazole; pH 7.4), and the protein was eluted with an elution buffer (20 mM Tris, 300 mM NaCl, and 250 mM imidazole; pH 7.4). The purified protein was concentrated using an amicon ultrafiltration concentrator (30kDa, Millipore, MA, USA). The protein concentration was determined by NanoDrop One spectrophotometer (Thermo Scientific, Walthamm, MA, USA).

### 4.4. The Electrophoretic Mobility Shift Assay

The natural compounds were purchased from Targetmol (Boston, MA, USA). Recombinant NGLY1 (2 μg) was incubated with a primary screening compound for 60 min at 37 °C, at which time denatured RNase B (New England Biolabs, Ipswich, MA, USA) (2 μg) was added. The mixture was incubated for 12–16 h at 37 °C before separation by SDS-PAGE (15%) and Coomassie staining. Further quantification was performed using Image J software. RNase B had a molecular weight of ~17 kDa. After NGLY1 mediated deglycosylation, the molecular weight of RNase B decreased to ~13.7 kDa. RNase B remained at ~17 kDa when NGLY1 activity was inhibited.

### 4.5. Toxicity Test

The safety profile of natural compounds was analyzed through calculation-based methods. ADMETlab 2.0 (https://admetmesh.scbdd.com (accessed on 10 October 2023)) was used to analyze the toxic effects of the NGLY1 inhibitors [33,34]. The ADMETlab 2.0 predicts the human hepatotoxicity (H-HT), drug induced liver injury (DILI), skin sensitization, carcinogenicity, eye corrosion, and eye irritation of the query compounds.

### 4.6. CCK-8 Analysis of Cell Viability Assay

HEK293T cells were cultured in Dulbecco’s modified Eagle’s medium (DMEM; Gibico, Shanghai, China) supplemented with 10% fetal bovine serum (FBS; Gibico, NSW, Australia) and 1% penicillin-streptomycin (Beyotime, Beijing, China) at 37 ℃ with 5% CO_2_. 5000 cells were seeded in each well of a 96-well plate. Cell viability was measured using the Cell Counting Kit-8 (Beyotime, Beijing, China) according to the manufacturer’s instructions.

## 5. Conclusions

In conclusion, NGLY1, an essential enzyme involved in the deglycosylation of misfolded glycoproteins through the ERAD pathway, has emerged as a pivotal enzyme with diverse therapeutic potential. Through a comprehensive structure-based virtual analysis of a natural compound library, and by validating the inhibitory effects of compounds using electrophoretic mobility shift assays, we identified three promising NGLY1 inhibitors: Poliumoside, Soyasaponin Bb, and Saikosaponin B2. These compounds were derived from traditional Chinese herbs with heat-clearing and detoxifying properties, displaying inhibitory activity against NGLY1. Preliminary toxicity predictions conducted through computer programs and analysis of cellular toxicity using the CCK-8 assays indicated that Soyasaponin Bb exhibited a higher safety profile in comparison to Z-VAD-FMK, while Poliumoside raised concerns regarding potential compound toxicity.

Moreover, it was revealed that the core structural motif shared by these inhibitors is a disaccharide structure featuring glucose and rhamnose. This structural insight suggested that their mode of action might involve binding to critical active sites of NGLY1, including Lys238 and Trp244, as they might serve as a basis for subsequent drug screening for NGLY1 inhibitors. Furthermore, natural compounds containing a disaccharide structure composed of glucose and rhamnose might also exert inhibitory effects on NGLY1.

Furthermore, NGLY1 inhibitors offer a multifaceted approach to address critical challenges in healthcare. They present a compelling solution to protease inhibitor resistance in blood cancer treatment and hold potential application in the management of melanoma. This study not only highlights the promise of Poliumoside, Soyasaponin Bb, and Saikosaponin B2 as lead compounds for NGLY1 inhibition but also provides a valuable foundation for future drug development efforts in the pursuit of effective antiviral and anticancer therapies.

## Figures and Tables

**Table 1:** Molecular docking information of 4 NGLY1 inhibitors.

Compound	Docking Score	Molecular Formula	Weight (g/mol)	Noncovalent Interactions	Amino Acid Sites								
Z-VAD-FMK	−4.560	C22H30FN3O7	467.494	4 H–bond	Lys238	/	/	/	/	Lys530	Val531	Thr533	
Poliumoside	−10.088	C35H46O19	770.734	1 Pi–pi 6 H–bond	Lys238	Glu239	Trp244	/	Trp369	/	Val531	/	Glu544
Soyasaponin Bb	−8.497	C48H78O18	943.134	3 H–bond	Lys238	/	Trp244	Glu356	/	/	/	/	/
Saikosaponin B2	−6.007	C42H68O13	780.993	5 H–bond	Lys238	Glu239	/	Glu356	/	/	/	/	/

**Table 2:** Predictive toxicity properties of NGLY1 inhibitors.

Target	H-HT	DILI	Skin Sensitization	Carcinogenicity	Eye Corrosion	Eye Irritation
Z-VAD-FMK	0.377 * (−)	0.924 (+++)	0.401 (−)	0.238 (−−)	0.004 (−−−)	0.01 (−−−)
Poliumoside	0.375 (−)	0.953 (+++)	0.971 (+++)	0.031 (−−−)	0.003 (−−−)	0.311 (−)
Soyasaponin Bb	0.178 (−−)	0.044 (−−−)	0.2 (−−)	0.025 (−−−)	0.003 (−−−)	0.008 (−−−)
Saikosaponin B2	0.281 (−−)	0.008 (−−−)	0.053 (−−−)	0.024 (−−−)	0.003 (−−−)	0.006 (−−−)

*: The value is the probability of being toxic. For the classification endpoints, the prediction probability values are transformed into six symbols: 0–0.1 (−−−), 0.1–0.3 (−−), 0.3–0.5 (−), 0.5–0.7 (+), 0.7–0.9 (++), and 0.9–1.0 (+++). H-HT: human hepatotoxicity; DILI: drug induced liver injury.

**Table 3:** PCR primer sequences for constructing NGLY1 mutants.

Mutant		Primer 5′-3′
K238D	Forward	CACCCAGTGAAAAAATTCTTCGTCAAACCAGTGCAAAAGCTCCAG
	Reverse	CTGGAGCTTTTGCACTGGTTTGACGAAGAATTTTTTCACTGGGTG
E239K	Forward	TCACCCAGTGAAAAAATTCTTTCTTAAACCAGTGCAAAAGCTC
	Reverse	GAGCTTTTGCACTGGTTTAAGAAAGAATTTTTTCACTGGGTGA
W244A	Forward	TGCTGCACAAAACGTTATTCACCGCGTGAAAAAATTCTTCCTTAAACC
	Reverse	GGTTTAAGGAAGAATTTTTTCACGCGGTGAATAACGTTTTGTGCAGCA
T533A	Forward	CCATGTGCCAGTCTGCTTCAACTTTTCTGAATATAGATTCCATT
	Reverse	AATGGAATCTATATTCAGAAAAGTTGAAGCAGACTGGCACATGG
D534K	Forward	CAAATATACCATGTGCCACTTTGTTTCAACTTTTCTGAATATAGATTCCATTTTCC
	Reverse	GGAAAATGGAATCTATATTCAGAAAAGTTGAAACAAAGTGGCACATGGTATATTTG

## Data Availability

The datasets used and/or analyzed during the current study are available from the corresponding author on reasonable request.

## Supplementary Materials

The following supporting information can be downloaded at: https://www.mdpi.com/article/10.3390/molecules28237758/s1. Refs [46,47,48,49,50,51,52,53,54,55,56,57,58,59,60,61,62] are cited in the Supplementary Material.
