# Supplementary material for: A Natural Compound Containing a Disaccharide Structure of Glucose and Rhamnose Identified as Potential N-Glycanase 1 (NGLY1) Inhibitors
Source: Molecules. 2023 Nov 24;28(23):7758. doi: 10.3390/molecules28237758 (PMC10707914; doi:10.3390/molecules28237758)
Supplement: Supplementary file 1 [file molecules-28-07758-s001.zip › molecules-2705372-supplementary.pdf]

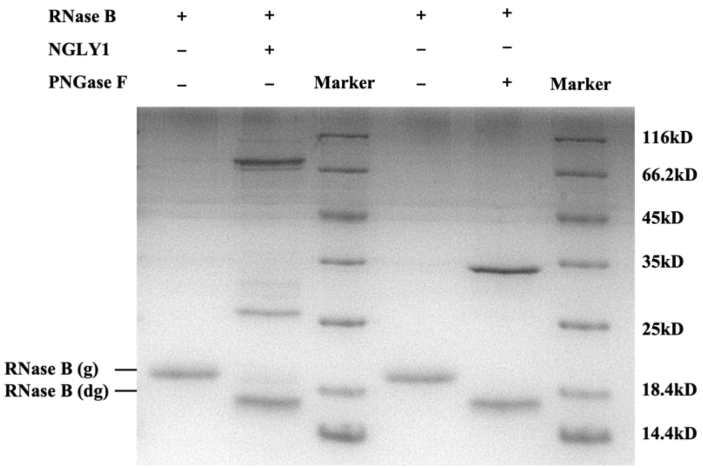

Figure S1. The enzyme effects of NGLY1 and PNGase F using the electrophoretic mobility shift assay.

Table S1. Information of 17 primary screening compounds.

Compound	CAS	Molecular Formula	Weight (g/mol)	2D Structure	Source	Docking Score	Bioactivity
Poliumoside	94079-81-9	C35H46O19	770.7	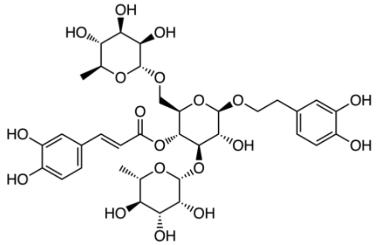	Brandisia hancei	-10.088	Related to glycosylation[1]
Soyasaponin Bb	51330-27-9	C48H78O18	943.1	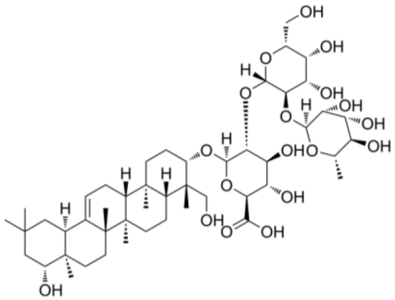	Glycine max	-8.497	Related to glycosylation[2]

Astragaloside IV	84687-43-4	C41H68O14	785	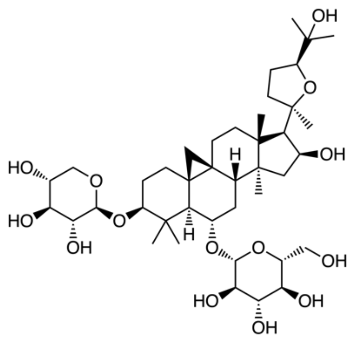	Astragalus membranaceus	-7.722	Anti-tumor[3]
Notoginsenoside R1	80418-24-2	C47H80O18	933.1	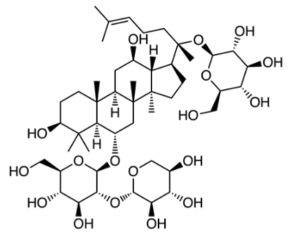	Panax notoginseng	-6.549	Anti-inflammatory and antioxidant[4]
Polyphyllin I	50773-41-6	C44H70O16	855	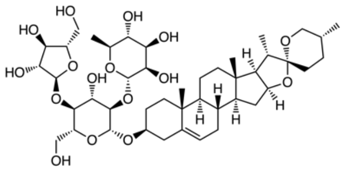	Paris	-6.52	Anti-tumor[5]
Amarogentin	21018-84-8	C29H30O13	586.5	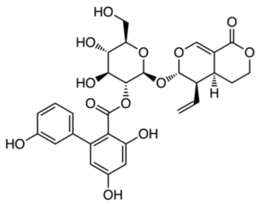	Gentiana scabra Bunge	-6.382	Anti-tumor and antioxidant[6]

Ginsenoside F2	62025-49-4	C42H72O13	785	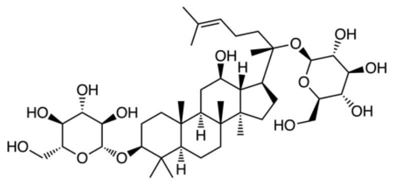	Panax ginseng	-6.367	Anti-tumor[7]
Loganin	18524-94-2	C17H26O10	390.4	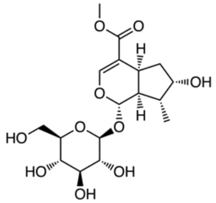	Strychnos nux-vomica	-6.301	Anti-inflammatory[8]
Lycorine	476-28-8	C16H17NO4	287.31	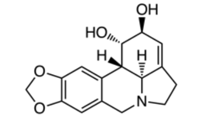	Lycoris radiata	-6.185	Anti-tumor[9]
Saikosaponin B2	58316-41-9	C42H68O13	781	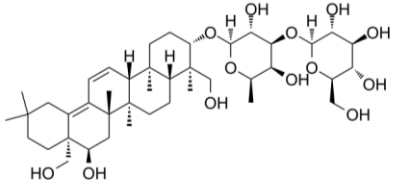	Bupleurum kaoi	-6.007	Anti-tumor[10]
Amygdalin	29883-15-6	C20H27NO11	457.4	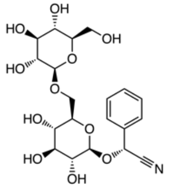	apricot kernel	-6.006	Anti-tumor[11]

Arctiin	20362-31-6	$C_{27}H_{34}O_{11}$	534.6	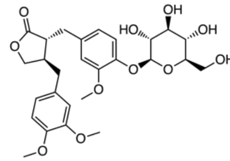	Arctium lappa	-5.938	Anti-tumor[12]
Vitexin	38953-85-4	$C_{21}H_{20}O_{10}$	432.4	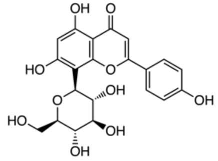	Vitex trifolia	-5.737	Anti-tumor, anti-inflammatory and antioxidant[13]
Nordihydroguaiaretic acid	500-38-9	$C_{18}H_{22}O_4$	302.4	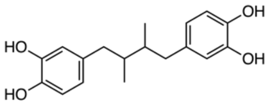	Guaiacum	-5.216	Antioxidant[14]
Camalexin	135531-86-1	$C_{11}H_8N_2S$	200.26	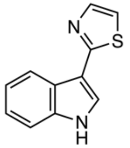	Arabidopsis	-5.024	Anti-tumor[15]
Aloin	1415-73-2	$C_{21}H_{22}O_9$	418.4	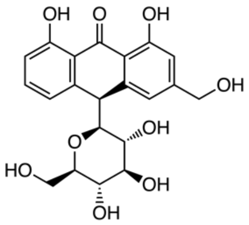	Aloe	-4.956	Anti-tumor[16]
3-Indolepropionic acid	830-96-6	$C_{11}H_{11}NO_2$	189.21	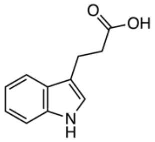	Animal	-4.826	Antioxidant[17]

Reference:

1. Yu, S. Y.; Lee, I. S.; Jung, S. H.; Lee, Y. M.; Lee, Y. R.; Kim, J. H.; Sun, H.; Kim, J. S., Caffeoylated phenylpropanoid glycosides from Brandisia hancei inhibit advanced glycation end product formation and aldose reductase in vitro and vessel dilation in larval zebrafish in vivo. *Planta Med* **2013,** 79, (18), 1705-9.
2. Lijie, Z.; Ranran, F.; Xiuying, L.; Yutang, H.; Bo, W.; Tao, M., Soyasaponin Bb Protects Rat Hepatocytes from Alcohol-Induced Oxidative Stress by Inducing Heme Oxygenase-1. *Pharmacogn Mag* **2016,** 12, (48), 302-306.
3. Jiang, K.; Lu, Q.; Li, Q.; Ji, Y.; Chen, W.; Xue, X., Astragaloside IV inhibits breast cancer cell invasion by suppressing Vav3 mediated Rac1/MAPK signaling. *Int Immunopharmacol* **2017,** 42, 195-202.
4. Li, H.; Zhu, J.; Xu, Y. W.; Mou, F. F.; Shan, X. L.; Wang, Q. L.; Liu, B. N.; Ning, K.; Liu, J. J.; Wang, Y. C.; Mi, J. X.; Wei, X.; Shao, S. J.; Cui, G. H.; Lu, R.; Guo, H. D., Notoginsenoside R1-loaded mesoporous silica nanoparticles targeting the site of injury through inflammatory cells improves heart repair after myocardial infarction. *Redox Biol* **2022,** 54, 102384.
5. Wu, L.; Li, Q.; Liu, Y., Polyphyllin D induces apoptosis in K562/A02 cells through G2/M phase arrest. *J Pharm Pharmacol* **2014,** 66, (5), 713-21.
6. Li, S.; Li, X.; He, F.; Jiao, R.; Zhang, S.; Li, Z., Amarogentin promotes osteoblast differentiation in oestrogen-deficiency-induced osteoporosis rats by modulating the Nrf-2/MAPK/ERK signalling pathway. *Arch Med Sci* **2023,** 19, (2), 452-457.
7. Siraj, F. M.; SathishKumar, N.; Kim, Y. J.; Kim, S. Y.; Yang, D. C., Ginsenoside F2 possesses anti-obesity activity via binding with PPARγ and inhibiting adipocyte differentiation in the 3T3-L1 cell line. *J Enzyme Inhib Med Chem* **2015,** 30, (1), 9-14.
8. Liu, S.; Shen, H.; Li, J.; Gong, Y.; Bao, H.; Zhang, J.; Hu, L.; Wang, Z.; Gong, J., Loganin inhibits macrophage M1 polarization and modulates sirt1/NF-κB signaling pathway to attenuate ulcerative colitis. *Bioengineered* **2020,** 11, (1), 628-639.
9. Li, C.; Deng, C.; Pan, G.; Wang, X.; Zhang, K.; Dong, Z.; Zhao, G.; Tan, M.; Hu, X.; Shi, S.; Du, J.; Ji, H.; Wang, X.; Yang, L.; Cui, H., Lycorine hydrochloride inhibits cell proliferation and induces apoptosis through promoting FBXW7-MCL1 axis in gastric cancer. *J Exp Clin Cancer Res* **2020,** 39, (1), 230.
10. Lin, L. T.; Chung, C. Y.; Hsu, W. C.; Chang, S. P.; Hung, T. C.; Shields, J.; Russell, R. S.; Lin, C. C.; Li, C. F.; Yen, M. H.; Tyrrell, D. L.; Lin, C. C.; Richardson, C. D., Saikosaponin b2 is a naturally occurring terpenoid that efficiently inhibits hepatitis C virus entry. *J Hepatol* **2015,** 62, (3), 541-8.
11. Song, Z.; Xu, X., Advanced research on anti-tumor effects of amygdalin. *J Cancer Res Ther* **2014,** 10 Suppl 1, 3-7.
12. Matsuzaki, Y.; Koyama, M.; Hitomi, T.; Yokota, T.; Kawanaka, M.; Nishikawa, A.; Germain, D.; Sakai, T., Arctiin induces cell growth inhibition

through the down-regulation of cyclin D1 expression. *Oncol Rep* **2008,** 19, (3), 721-7.
13. Jang, S. A.; Hwang, Y. H.; Yang, H.; Ryuk, J. A.; Gu, D. R.; Ha, H., Ethanolic extract of Pyrrosia lingua (Thunb.) Farw. ameliorates OVX-induced bone loss and RANKL-induced osteoclastogenesis. *Biomed Pharmacother* **2022,** 147, 112640.
14. Manciu, F. S.; Guerrero, J.; Bennet, K. E.; Chang, S. Y.; Rahman, M.; Martinez Lopez, L. V.; Chantigian, S.; Castellanos, M.; Manciu, M., Assessing Nordihydroguaiaretic Acid Therapeutic Effect for Glioblastoma Multiforme. *Sensors (Basel)* **2022,** 22, (7).
15. Yamashita, N.; Taga, C.; Ozawa, M.; Kanno, Y.; Sanada, N.; Kizu, R., Camalexin, an indole phytoalexin, inhibits cell proliferation, migration, and mammosphere formation in breast cancer cells via the aryl hydrocarbon receptor. *J Nat Med* **2022,** 76, (1), 110-118.
16. Tan, C.; Zhu, W.; Lu, Y., Aloin, cinnamic acid and sophorcarpidine are potent inhibitors of tyrosinase. *Chin Med J (Engl)* **2002,** 115, (12), 1859-62.
17. Cao, B.; Zhao, R. Y.; Li, H. H.; Xu, X. M.; Cui, H.; Deng, H.; Chen, L.; Wei, B., Oral administration of asparagine and 3-indolepropionic acid prolongs survival time of rats with traumatic colon injury. *Mil Med Res* **2022,** 9, (1), 37.
